# Permissible Area Analyses of Measurement Errors with Required Fault Diagnosability Performance

**DOI:** 10.3390/s19224880

**Published:** 2019-11-08

**Authors:** Dong-Nian Jiang, Wei Li

**Affiliations:** 1 College of Electrical and Information Engineering, Lanzhou University of Technology, Lanzhou 730050, China; liwei@lut.cn; 2 Key Laboratory of Gansu Advanced Control for Industrial Processes, Lanzhou 730050, China

**Keywords:** fault diagnosability, quantitative evaluation, Kullback–Leibler divergence

## Abstract

Fault diagnosability is the basis of fault diagnosis. Fault diagnosability evaluation refers to whether there is enough measurable information in the system to support the rapid and reliable detection of a fault. However, due to unavoidable measurement errors in a system, a quantitative evaluation index of system fault diagnosability is inadequate. In order to overcome the adverse effects of measurement errors, improve the accuracy of the quantitative evaluation of fault diagnosability, and improve the safety level of the system, a method for a permissible area analysis of measurement errors for a quantitative evaluation of fault diagnosability is proposed in this paper. Firstly, in order for the residuals obey normal distribution, a design method of the permissible area of measurement errors based on the Kullback–Leibler divergence (KLD) is given. Secondly, two key problems in calculating the KLD are solved by sparse kernel density estimation and the Monte Carlo method. Finally, the feasibility and validity of the method are analyzed through a case study.

## 1. Introduction

With the increasing scale of modern engineering, the safety and reliability of a system has become a research hotspot, which has led to the rapid development of fault detection, fault isolation, and fault prediction [1,2]. At present, the research into these security technologies is to study the security of a system after its design, e.g., fault diagnosis, aiming at the possible conditions of a fault. However, the research on the performance of fault diagnosis at the beginning of the design of a system is insufficient.

Fault diagnosability is the basis of fault diagnosis, including the evaluation and design of fault diagnosability. The current research idea is that before the system design, the fault diagnosability should be evaluated first. Only when achieving a certain value of some fault diagnosability evaluation index can the system have the ability to diagnose possible faults. If the evaluation of the fault diagnosability shows that it does not meet the design requirements, it is necessary, within the system design process, to ensure the fault diagnosability of the system. The fault diagnosability evaluation index can be improved by adding information about the measuring points in the system. Therefore, the ability to diagnosis faults should be incorporated into the design framework as an intrinsic characteristic of the system. [3] elaborates in detail the problem of fault diagnosability evaluation for a system. In [4,5,6], the methods of polynomial basis, equivalent space, and coprime decomposition are used to study the fault diagnosability evaluation of a system. However, most of the current methods for fault diagnosability evaluation are based on qualitative research. Due to the influence of uncertain factors such as measurement errors and interference, it is difficult for a qualitative description to accurately describe fault diagnosis ability. [7] quantitatively evaluates the fault diagnosability of linear systems by introducing a fault model, which provides a new idea for the study of fault diagnosability. It is found that fault diagnosability is an inherent property of the system. Therefore, in order to avoid the inaccuracy of a model-based fault diagnosability algorithm, it is more practical to make a quantitative evaluation of fault diagnosability by acquiring system measurement data.

It can be seen that measurement data is the basis of a quantitative evaluation of fault diagnosability. However, due to the unavoidable influence of noise, interference, recording errors, non-ideal instruments, and other factors in the measurement process, it is difficult to accurately obtain the data needed to evaluate the fault diagnosability of the system in reality. In [8], the method of sensor accuracy compensation is given to solve the problem of sensor measurement error. In [9], a method for the safety evaluation of device noise is presented for the comparison of degradation curves between ideal and non-ideal devices. The influence of measurement errors on system performance degradation is given in [10,11], which provides some inspiration for fault diagnosability evaluation. Therefore, we have to consider the following questions: (1) Can the quantitative evaluation of fault diagnosability for measurement data affected by random measurement errors meet the performance requirements of fault diagnosability evaluation? (2) On the premise of determining the evaluation index of fault diagnosability, what is the availability of measurement data? (3) How does one determine the permissible area of measurement errors in measurement data to avoid degradation of fault diagnosable performance indicators? All of the above problems are obstacles that need to be faced when evaluating the diagnosability of system faults. However, most of the current research is to evaluate fault diagnosability directly after acquiring the measured data, which is a forward problem. When there are errors in the measured data, it is easy to cause a deviation of the evaluation results. Such an evaluation index with deviation will inevitably lead to mistakes in the fault diagnosis. Therefore, on the premise of a quantitative evaluation index of fault diagnosability evaluation, it is necessary to determine the permissible area of the measurement errors. This is the key problem to be solved for an application-oriented quantitative evaluation of fault diagnosability.

Based on this, this paper considers the determination of the feasible region of the measurement errors in fault diagnosability evaluation. By using a difference measurement method based on the Kullback–Leibler divergence (KLD), with the help of sparse kernel density estimation and the Monte Carlo method, the permissible area of measurement errors can be pre-positioned before fault diagnosability evaluation. The main contribution of this paper is not only to obtain the quantitative evaluation index of fault diagnosability, but also to consider the impact of measurement errors on the diagnosability, which provides a reference for the design of the system based on security. 

The remainder of this paper is organized as follows. Section 2 presents the problem of the permissible area of measurement errors. Section 3 discusses the design of the permissible area of measurement errors. The quantitative evaluation of fault diagnosability under measurement errors is introduced in Section 4. Section 4 employs a continuous stirred tank reactors (CSTRs) system model to demonstrate the permissible area design of different fault models. Finally, some conclusions are given in Section 5.

## 2. Problem Description

In order to facilitate the discussion of the problem, we consider a non-linear dynamic process:

(1) $$\begin{array}{l} \dot{x}=g(x,u,f)\\ y=h(x,u,w) \end{array}$$

where $x\in R^{n}$ is the state vector of the system, $y\in R^{m}$ is the output of the system, $u\in R^{q}$ is the input function, g and h are non-linear functions, w is the measurement error with a known probability density function (PDF), and f is the system fault or sensor fault.

In order to evaluate the security performance of such a non-linear dynamic process, the usual method is to obtain information about the residuals of the system first, and then extract the effective information contained in the residuals to judge the security situation faced by the system. Here, we can obtain the residuals by comparing the measurements y with the estimates $\hat{y}$:

(2) $$r=y-\hat{y}$$

The residuals are the basis for assessing the safety of a system. Taking the fault diagnosis of the system as an example, the residuals can not only detect faults and judge whether the system is in failure, but also estimate and locate a fault through the feature extraction of the residuals and then effectively isolate the fault.

Under ideal conditions, if the system is not affected by measurement errors such as interference and noise, and no performance degradation or failure occurs, the residuals should be zero. In the case of a diagnosable fault, the occurrence of the fault will inevitably cause the residuals to deviate from zero. Although the specific forms of deviation vary, it is sufficient to determine whether this fault situation can be detected. Of course, in the case of a fault that does not satisfy diagnosability, the fault can not be reflected in the residuals, since there is insufficient measurement information. It is necessary to add the measurement information to the system in order to make it diagnosable. We will not discuss this here. 

However, the reality is that even if the system does not fail, it will inevitably be affected by various measurement errors, which will make the residuals deviate from zero and fluctuate with the influence of the uncertainties. That is to say, the residuals r differ from zero and fluctuate even if the system does not fail. Hence, it can be found that the uncertainty of the system will have an impact on the study of fault diagnosability evaluation.

If we only consider the measurement errors caused by noise, interference, recording errors, and non-ideal instruments in the measurement process, the PDF of the residuals r should be similar to the PDF of the measurement errors w in the absence of a system failure, according to the analysis in [7]. On the basis of this theory, when the PDF of r deviates from the PDF of w, the system may fail, which can be used as a theoretical basis for judging whether the system has fault diagnosability. For different faults with fault diagnosability, the difference between the PDFs of their residuals can be used as the theoretical basis for fault isolability.

However, whether the above theory can be effectively carried out depends on whether the measurement errors and fault can be accurately distinguished from the residuals. It is not difficult to see that an increase in measurement errors will undoubtedly increase the difficulty of separating faults from the residuals, thus directly leading to a misjudgment of the fault diagnosability. Moreover, although a small fault may exist in the system, because of its small amplitude and low frequency, can easily be submerged in the measurement errors of the system, which makes the diagnosability evaluation index of the fault low. It will further increase the design cost of fault diagnosability and reduce the efficiency and reliability of fault diagnosis.

In view of the above considerations, in order to ensure the effective evaluation of fault diagnosability, it is necessary to reduce the deviation of fault diagnosability evaluation and determine the permissible area of measurement errors.

## 3. Permissible Area of Measurement Errors for Evaluation of Fault Diagnosability

### 3.1. Analysis of the Residuals of the Measurement Errors in Different Domains

**Figure 1:**
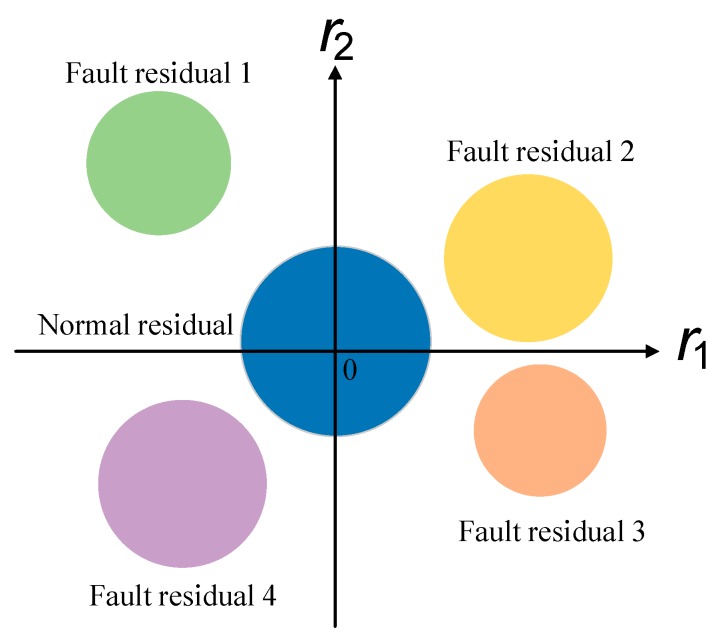
Residual distributions without measuring errors.

Through the above analysis, we can see that fault diagnosability can be evaluated by the residuals. Figure 1 shows a two-dimensional distribution of the residuals without measurement errors, in which it is assumed that the residuals of a normal system and of the system in fault are located in differently colored regions. There are many methods available to evaluate the diagnosability of the system shown in Figure 1. For example, the Euclidean distance in [12,13] can determine the diagnosability by the distance between the state variables. In addition, the Mahalanobis distance used in [14,15,16,17] and the improved support vector machine method proposed in [18,19] can be used to evaluate the diagnosability by introducing state-to-state distances.

**Figure 2:**
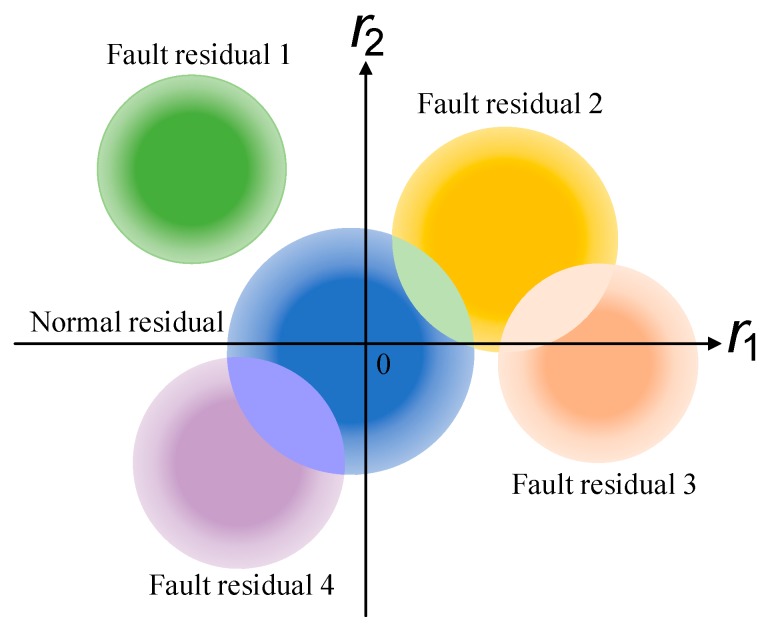
Residual distributions with measurement errors.

In view of the unavoidable uncertainty in an actual system, if measurement errors are introduced, the residuals are as shown in Figure 2.

As can be seen from Figure 2, the existence of measurement errors enlarges the range of the residuals. If the range of measurement errors is not limited, it might easily happen that different residuals overlap. In Figure 2, both residuals between fault mode 2 and fault mode 4 overlap with the residuals of the normal mode, and the residuals of fault mode 2 and fault mode 3 overlap. This data crossover makes it difficult to distinguish the diagnosability of a fault. The aforementioned similarity evaluation methods, such as Euclidean distance, will lead to evaluation errors because of a large number of data duplications, and the existence of outliers will also cause misjudgments by these evaluation methods.

In fact, from the thermal gradient characteristics of the data colors in Figure 2, we can see that, first of all, although the residuals overlap due to the existence of measurement errors, most of the data are still concentrated in the noise-free data range. Secondly, in order to classify the residuals shown in Figure 2 and determine the diagnosability of the system, the traditional method based on the distance between the state is difficult to exploit. Moreover, the overlap of residuals will be more serious if the measurement errors increase or the fault amplitude is smaller. Therefore, it is necessary to limit the range of measurement errors to ensure that the fault can be detected and isolated.

### 3.2. Permissible Area Analyses of Measurement Errors Based on Fault Detectability

In order to remedy the shortcomings of the traditional measurements of distance, a permissible area design method for measuring noise based on the Kullback–Leibler divergence (KLD) is introduced in this paper. Kullback–Leibler divergence, also known as relative entropy, was originally proposed by Kullback and Leibler in 1951. This method is usually used as a measure of the similarity of two probability distributions and is widely used in statistics and pattern recognition. Let h(x) and g(x) define two PDFs on space ${\mathbb{R}}^{d}$, where ${\mathbb{R}}^{d}$ is the characteristic dimension. Then, the KLD between h(x) and g(x) can be defined as:

(3) $$K(h∥g)=\int_{{\mathbb{R}}^{d}}h(x)\log\frac{h(x)}{g(x)}dx$$

The significance of Equation (3) is that for given PDFs h(x) and g(x), the difference is characterized by the KLD. It has the following three attributes:

(1) Self-similarity: K(h∥h)=0;
(2) Self-identity: h=g if and only if K(h∥g)=0;
(3) Nonnegativity: for all h(x) and g(x), K(h∥g)≥0.

It is assumed that the residuals satisfy the normal distribution, with PDF $p_{NF}∼N(\mu_{0},\sigma_{0}^{2})$ when the system is normal. Then, the problem of determining the permissible area of the measurement errors is to determine the parameters $\mu_{0}$ and $\sigma_{0}$. If the PDF of the residuals is $p_{i}$ when fault $f_{i}$ occurs, the difference between the two PDFs can be evaluated by the formula:

(4) $$K(p_{i}\|p_{NF})=\int_{-\infty }^{\infty }p_{i}(r)\log\frac{p_{i}(r)}{p_{NF}(r)}dr$$

When there are no measurement errors in the system, the residuals can be detected sensitively by introducing Formula (4), which ensures the detectability of a system fault. However, due to the unavoidable measurement errors in the system, when the area of the measurement errors increases, the signal-to-noise ratio will inevitably decrease, which will result in the decrease of the detectability evaluation index (4).

Therefore, in order to achieve an evaluation index of fault detectability, the minimum evaluation index of fault detectability should be given as $\delta_{d}$ firstly. On the basis of obtaining the minimum evaluation index, the permissible area of measurement errors can be discussed. It can be seen that fault diagnosability should be satisfied, i.e.:

(5) $$K(p_{i}\|p_{NF})=\int_{-\infty }^{\infty }p_{i}(r)\log\frac{p_{i}(r)}{p_{NF}(r)}dr\geq \delta_{d}$$

If the residuals *r* obey the normal distribution, the following theorem can be used to simplify the calculation.

**Theorem** **1:** *If*m(x)*and*n(x)*obey the*l-*dimensional normal distribution*$N(U_{1},V_{1})$*and*$N(U_{2},V_{2})$*respectively, their relative entropy is*:

(6) $$K(m\|n)=\frac{1}{2}\{\log\frac{\left|V_{2}\right|}{\left|V_{1}\right|}+Tr(V_{1}(V_{2}^{-1}-V_{1}^{-1})+{\left(U_{1}-U_{2}\right)}^{T}V_{2}^{-1}(U_{1}-U_{2}))\}$$

**Proof:**  From the concept of relative entropy, we can get:

$$\begin{array}{ll} K(m\|n) & =\int_{R^{n}}m(x)\log\frac{m(x)}{n(x)}dx\\ & =\int_{R^{n}}m(x)\log m(x)dx-\int_{R^{n}}m(x)\log n(x)dx\\ & =-\frac{l}{2}\log2\pi -\frac{1}{2}\log\left|V_{1}\right|-\frac{1}{2}Tr(V_{1}V_{1}^{-1})+\frac{l}{2}\log2\pi \\ & \;\;\;+\frac{1}{2}\log\left|V_{2}\right|+\frac{1}{2}Tr(V_{1}V_{2}^{-1})+\frac{1}{2}\{{\left(U_{1}-U_{2}\right)}^{T}V_{2}^{-1}(U_{1}-U_{2})\}\\ & =\frac{1}{2}\{\log\frac{\left|V_{2}\right|}{\left|V_{1}\right|}+Tr(V_{1}(V_{2}^{-1}-V_{1}^{-1})+{\left(U_{1}-U_{2}\right)}^{T}V_{2}^{-1}(U_{1}-U_{2}))\} \end{array}$$

□

Thus, the acquisition method of the permissible area of measurement errors can be obtained, as shown in Corollary 1.

**Corollary** **1:** *If the residuals r obey the normal distribution, i.e.,*$p_{i}∼N(\mu_{1},\sigma_{1}^{2})$*and*$p_{NF}∼N(\mu_{0},\sigma_{0}^{2})$*, the permissible area of measurement errors based on the given fault detectability evaluation index*$\delta_{d}$*can be achieved by*:

(7) $$\frac{1}{2}\{\log\frac{\sigma_{1}^{2}}{\sigma_{0}^{2}}+\frac{\sigma_{0}^{2}}{\sigma_{1}^{2}}-1+\frac{{\left(\mu_{1}-\mu_{0}\right)}^{2}}{\sigma_{1}^{2}}\}\geq \delta_{d}$$

The above calculation method can not only simplify the calculation, but also expand the measurement scale of the difference.

### 3.3. Permissible Area Analyses of Measurement Errors Based on Fault Isolability

Consider two different faults $f_{i}$ and $f_{j}$ that may occur in the system. Assuming that the PDFs of the residuals are $p_{i}$ and $p_{j}$, the degree of difference can be evaluated by the formula:

(8) $$K(p_{i}\|p_{j})=\int_{-\infty }^{\infty }p_{i}(r)\log\frac{p_{i}(r)}{p_{j}(r)}dr$$

Fault isolation is a very complex problem. Since there is the possibility of multiple component faults corresponding to one abnormal symptom in the system, it is difficult to isolate faults effectively. Moreover, due to the influence of measurement errors in the system, the range of residuals will be expanded, and even the overlap of residuals under different faults will occur, which makes the research of fault isolability difficult. In order to improve the evaluation index of fault isolability, it is necessary to analyze the permissible area of measurement errors. It is assumed that for two different faults $f_{i}$ and $f_{j}$, the minimum allowable value of the difference is $\delta_{i}$, then it should be satisfied that:

(9) $$K(p_{i}\|p_{j})=\int_{-\infty }^{\infty }p_{i}(r)\log\frac{p_{i}(r)}{p_{j}(r)}dr\geq \delta_{i}$$

If the PDFs of the residuals under faults $f_{i}$ and $f_{j}$ obey the normal distribution, Theorem 1 can also be used to simplify the calculation, as shown in Corollary 2.

**Corollary** **2:** *If the residuals r obey the normal distribution, i.e., if*$p_{i}∼N(\mu_{1},\sigma_{1}^{2})$*and the permissible area of measurement errors satisfies Equation (10) on the basis of a given fault isolability evaluation index*$\delta_{i}$:

(10) $$\frac{1}{2}\{\log\frac{\sigma_{1}^{2}}{\sigma_{2}^{2}}+\frac{\sigma_{2}^{2}}{\sigma_{1}^{2}}-1+\frac{{\left(\mu_{1}-\mu_{2}\right)}^{2}}{\sigma_{1}^{2}}\}\geq \delta_{i}$$

Through the above analysis, the permissible area of measurement errors can be obtained on the basis of the given performance indicators of fault detectability and isolability. However, in the process of solving Equations (5) and (9), there are still several difficulties. First, the PDF of the residuals needed in the calculation process is unknown if the PDFs of the residuals don’t obey the normal distribution. Even assuming that the PDFs of the residuals obey the normal distribution, its mean and variance can not be determined, which makes the calculation of the measurement errors domain difficult. Second, it is difficult to calculate the KLD due to the non-linear structure of Equations (5) and (9). Therefore, it is necessary to design algorithms to overcome these two key problems in the isolability analysis of measurement errors. 

### 3.4. Key Problems in Permissible Area Analysis of Measurement Noise

For the estimation of the residual PDF, the traditional method is the kernel density estimator (KDE) method, but in order to ensure the accuracy of the estimation of the PDF, we usually use a large number of data points to carry out the estimation. Therefore, a method based on sparse kernel density estimator (SKDE) [20] seems to be more suitable. This method has a fast calculation speed, small memory requirement, and yields a more smooth and accurate estimation of the PDF.

Assuming that there are *N* points in the given residuals, whose set is $D_{N}={\left\{r_{i}\right\}}_{i=1}^{N}$, for $r_{i}\in R^{m}$ in the residual set, the location of the data points can be determined, but the PDF is not known. Our approach is: first, random sampling of data set $D_{N}$ to form a new data set $D_{M}=\{r_{1}^{\prime },r_{2}^{\prime },\cdots ,r_{M}^{\prime }\}$, which satisfies M<N. Then, the PDF p(r) can be obtained based on the kernel probability density estimation method as follows:

(11) $${\hat{p}}^{\left(M\right)}(r,\beta_{M},\sigma_{M})=\sum_{i=1}^{M}\beta_{i}K_{\sigma_{i}}(r,r_{i}^{\prime })$$

where $K_{\sigma_{i}}(r,r_{i}^{\prime })$ is the kernel of the PDF estimation, which is Gaussian. The central vector of the kernel is $r_{i}^{\prime }$, and the width of the kernel can be adjusted to $\sigma_{i}$.

(12) $$K_{\sigma_{i}}(r,r_{i}^{\prime })=\frac{1}{{\left(2\pi \sigma_{i}^{2}\right)}^{m/2}}\exp\left(-\frac{\|r-r_{i}^{\prime }{\|}^{2}}{2\sigma_{i}^{2}}\right)$$

where $\beta_{i}$ is the weight of the *i*th kernel, $\sigma_{M}={\left[\sigma_{1},\sigma_{2},\cdots ,\sigma_{M}\right]}^{T}$, $\beta_{M}={\left[\beta_{1},\beta_{2},\cdots ,\beta_{M}\right]}^{T}$, and $\beta_{M}^{T}l_{M}=1$, where $l_{M}$ is the *M*-dimensional column vector of all 1 values.

If ${\hat{z}}^{\left(l\right)}(r)$ represents the probability density estimate of step *l*, that is:

(13) $${\hat{z}}^{\left(l\right)}(r)=\sum_{i=1}^{l}\beta_{i}^{\left(l\right)}K_{\sigma_{i}}(r,r_{i}^{\prime }))$$

and $\sigma_{l}={\left[\sigma_{1},\sigma_{2},\cdots ,\sigma_{l}\right]}^{T}$, $\beta_{l}={\left[\beta_{1},\beta_{2},\cdots ,\beta_{l}\right]}^{T}$, p(r) can be estimated by using the following algorithm.

(1) Step 1, because $\beta_{1}^{\left(1\right)}=1$, set:

(14) $${\hat{z}}^{\left(1\right)}(r)=K_{\sigma_{1}}(r,r_{1}^{\prime })$$

(2) In step l, when l≥2, p(r) can be estimated in the following way:

(15) $${\hat{z}}^{\left(l\right)}(r)=\lambda_{l}{\hat{z}}^{\left(l-1\right)}(r)+(1-\lambda_{l})K_{\sigma_{l}}(r,r_{l}^{\prime })$$

where the width of the kernel $K_{\sigma_{l}}(r,r_{l}^{\prime })$ can be obtained by changing the width of the kernel from $\sigma_{0}$ to $\sigma_{l}$. It satisfies $0\leq \lambda_{l}\leq 1$, and $\lambda_{1}=0$.

By using the SKDE method mentioned above, the estimation of the residual PDF p(r) can be obtained, and the first key problem can be solved. For Equations (5) and (9) of the non-linear structure, we can use the Monte Carlo method to approximate the solution. 

Taking the solving process of Equation (9) as an example, by using the Monte Carlo method, we get the result:

(16) $$\hat{K}(p_{i}\|p_{j})=\frac{1}{n_{s}}\sum_{i=1}^{n_{s}}\log\frac{{\hat{p}}_{i}}{{\hat{p}}_{j}}$$

where $n_{s}$ is the number of ${\left\{z_{i}\right\}}_{1}^{n_{s}}$ sampled from ${\hat{p}}_{i}$. According to [21], the error in the estimation process of Formula (16) usually obeys the normal distribution, with expectation 0 and variance:

(17) $$\sigma_{MC}^{2}=\frac{1}{n_{s}}\left(E{\left[\log\left(\frac{{\hat{p}}_{i}}{{\hat{p}}_{j}}\right)\right]}^{2}\right)$$

That is, the estimation error $\tilde{r}\sim N(0,\sigma_{MC}^{2})$. By increasing $n_{s}$, through Equation (17) we can see that the variance of the Monte Carlo estimation process will also be reduced. 

**Figure 3:**
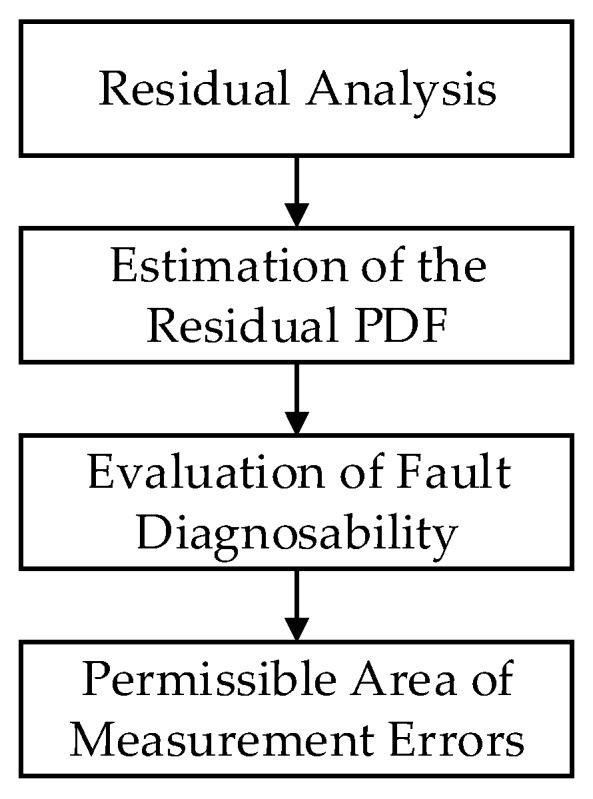
Schematic diagram of permissible area of measurement errors.

Thus, given the fault detectability index $\delta_{d}$ and fault isolability index $\delta_{i}$, the parameters $\mu_{0}$ and $\sigma_{0}$ corresponding to the permissible area of measurement errors can be determined. Generally speaking, small values of $\delta_{d}$ and $\delta_{i}$ correspond to the high requirements of fault diagnosability evaluation. The schematic diagram of the permissible area of measurement errors is shown in Figure 3.

## 4. Simulation

### 4.1. Non-Constant Temperature Continuous Stirred Tank Reactors (CSTRs)

**Figure 4:**
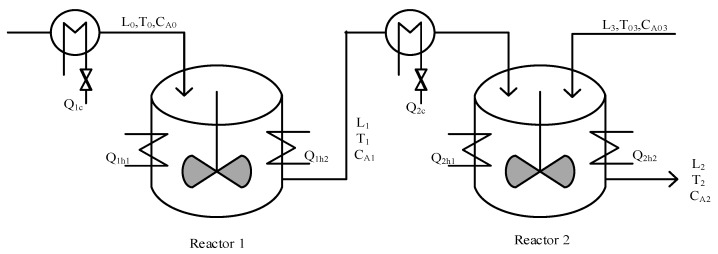
Schematic of continuous stirred tank reactors (CSTRs).

Referring to the system model in [22,23], the simulation object used in this paper is a CSTR, as shown in Figure 4. Three irreversible basic heating reactions $A\overset{K_{1}}{\to }B,$ $A\overset{K_{2}}{\to }U,$ $A\overset{K_{3}}{\to }R$ take place in the reactors. Here, A is the reactant and B is the expected product, while *U* and *R* are by-products. Reagent is added in reactor 1, its flow rate is $L_{0}$, its concentration is $C_{A0}$, and its temperature is $T_{0}$. The additive in reactor 2 is the product of reactant A and reactor 1. The flow rate of A is $L_{3}$, the concentration of A is $C_{A03}$, and the temperature is $T_{03}$. Due to the non-constancy of the temperatures of the reactors, $Q_{1h1}$ and $Q_{2h2}$ are used to regulate the temperature of the reactors. According to the conservation of material and energy, the mathematical model of the reactor can be obtained as follows.

(18) $$\begin{array}{l} \frac{dT_{1}}{dt}=\frac{L_{0}}{V_{1}}(T_{0}-T_{1})+\sum_{i=1}^{3}\frac{\left(-\Delta H_{i}\right)}{\rho c_{p}}R_{i}(C_{A1},T_{1})+\frac{Q_{1}}{\rho c_{p}V_{1}}\\ \frac{dC_{A1}}{dt}=\frac{L_{0}}{V_{1}}(C_{A0}-C_{A1})-\sum_{i=1}^{3}R_{i}(C_{A1},T_{1})\\ \frac{dT_{2}}{dt}=\frac{L_{1}}{V_{2}}(T_{1}-T_{2})+\frac{L_{3}}{V_{2}}(T_{03}-T_{2})+\sum_{i=1}^{3}\frac{\left(-\Delta H_{i}\right)}{\rho c_{p}}R_{i}(C_{A2},T_{2})+\frac{Q_{2}}{\rho c_{p}V_{2}}\\ \frac{dC_{A2}}{dt}=\frac{L_{1}}{V_{2}}(C_{A1}-C_{A2})+\frac{L_{3}}{V_{2}}(C_{A03}-C_{A2})-\sum_{i=1}^{3}R_{i}(C_{A2},T_{2}) \end{array}$$

where $R_{i}(C_{Aj},T_{j})=k_{i0}*\exp(-E_{i}/RT_{j})*C_{Aj}$, (*j* = 1,2), $T,C_{A},Q,V$ represent the reactor temperature, concentration of reactant *A*, reactor heat transfer ratio, and reactor volume, $\Delta H_{i},k_{i},E_{i}(i=1,2,3)$ represent the heat content, exponential constant, and reaction trigger energy respectively, while $c_{p}$ and ρ represent the reactor heat capacity and fluid density. At this time, the state variable of CSTRs is $X={\left[x_{1},x_{2},x_{3},x_{4}\right]}^{T}={\left[T_{1},C_{A1},T_{2},C_{A2}\right]}^{T}$. We take the measurement output of the system as y=CX,C=[1,1,1,1]. When the measurement output of the system is $\hat{y}$ in normal operation, the residual of the system is $r=y-\hat{y}$. The parameters of the reactor in normal operation are shown in Table 1. 

### 4.2. Estimation of Residual PDFs under Different Faults

By analying the possible faults of non-constant temperature CSTRs, the obtained failure scenarios are shown in Table 2.

**Figure 5:**
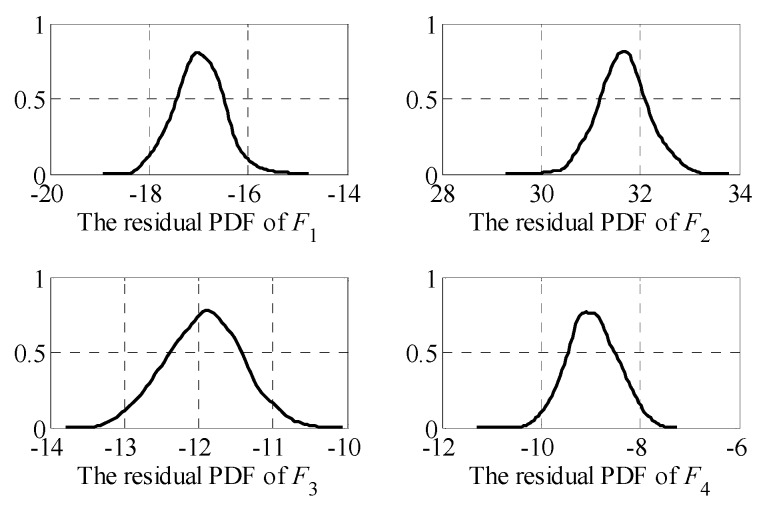
Estimation of residual probability density function (PDF).

Without considering the influence of measurement errors, the sparse kernel density estimation method has been used to estimate the residual PDFs for four possible fault modes. The estimation results are shown in Figure 5.

### 4.3. Quantitative Evaluation of Fault Diagnosability without Measuring Errors

On the basis of the above process, four possible fault modes of the system are quantitatively evaluated for fault detectability and isolability, and the evaluation results are shown in Table 3.

From Table 3, it can be seen that: (1) all four fault modes are detectable and their quantified evaluation results are different in size: the quantified evaluation results of detectability are $F_{1}>F_{2}>F_{3}>F_{4}$; (2) Although the difficulty of separating the four fault modes is different, they all have fault isolability; (3) Due to the asymmetry in the KLD calculation, the separable results show asymmetric structure; (4) Among them, faults $F_{4}$ and $F_{2}$ have the strongest isolability, while faults $F_{3}$ and $F_{4}$ have the weakest isolability.

### 4.4. Quantitative Evaluation of Fault Diagnosability under Measurement Errors

**Figure 6:**
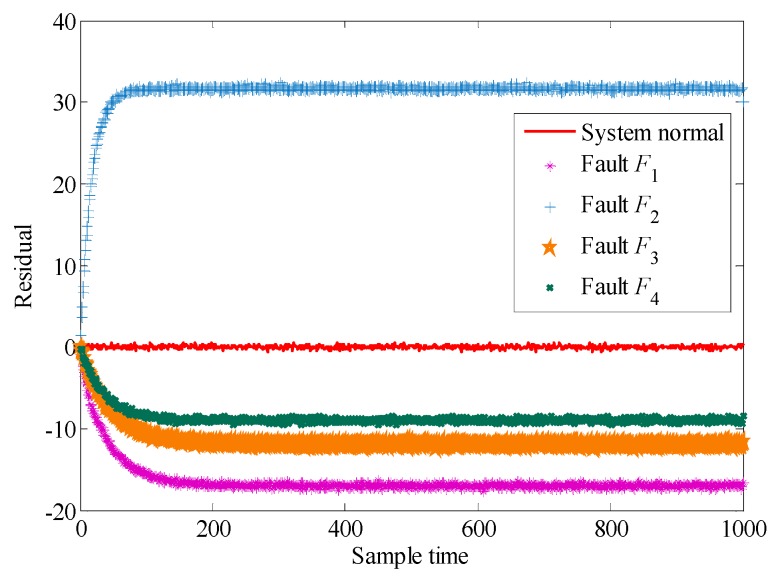
Residual curve under measurement errors.

Although the system has better fault detectability and fault isolability in Table 3, the influence of measurement errors is not considered. Assuming that the measurement errors of the system obey the normal distribution and satisfy w~N(0,0.2), the residuals are shown in Figure 6. 

It can be seen intuitively that even under the influence of measurement errors, the residual under normal and fault conditions still have better separation characteristics. Since the residuals of the different faults in Figure 6 are quite different, the traditional Euclidean distance can be used to evaluate the diagnosability of faults.

**Figure 7:**
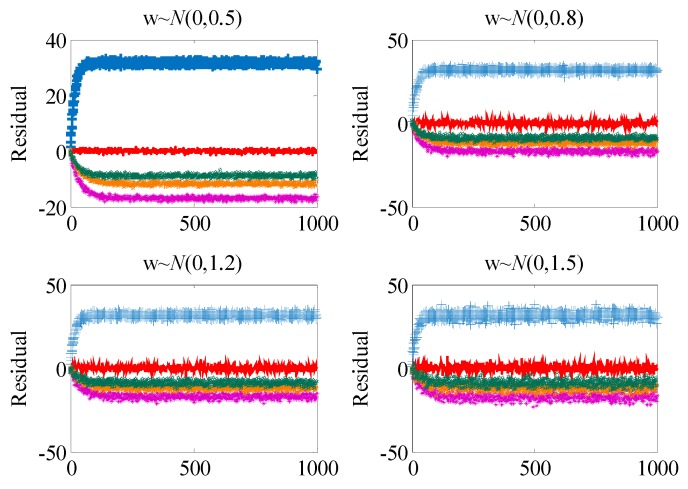
Residual curves under different measurement errors.

Figure 7 shows the residual curves under different measurement errors. It can be seen from the figure that with the increase of variance of measurement errors, the residual curves of fault $F_{1}$, $F_{3}$, and $F_{4}$ overlap, which makes it difficult to isolate the faults from each other and reduces the evaluation index of the fault isolability of the system. 

### 4.5. Permissible Area Analysis of Measuring Errors

It can be seen that in order to guarantee the achievement of a specified value of the evaluation index of fault diagnosability, the range of measurement errors of the system must be limited. Here, we give the minimum allowable values of the evaluation indexes of fault detectability and isolability $\delta_{d}=1.0$ and $\delta_{i}=1.0$. Thus, the permissible area of measurement errors can be designed by the method given in this paper, and the parameters of the measurement errors can be determined, as shown in Table 4. As can be seen from Table 4, with an increasing variance of measurement errors, the index of fault isolability decreases gradually. A change in the mean value of the measurement errors has a limited influence on the evaluation index of isolability, but it can be verified by experiments that the change has a great influence on the stability of the system: an increase of the mean value will lead to the instability of the system. When $\mu_{0}=0$, an increase of the variance to $\sigma_{0}=1.5$ will cause the isolability of faults $F_{3}$ and $F_{4}$ to be lower than the threshold. When $\mu_{0}=0.5$, a variance of $\sigma_{0}=1.2$ or more will make the faults $F_{3}$ and $F_{4}$ lose their isolability.

**Figure 8:**
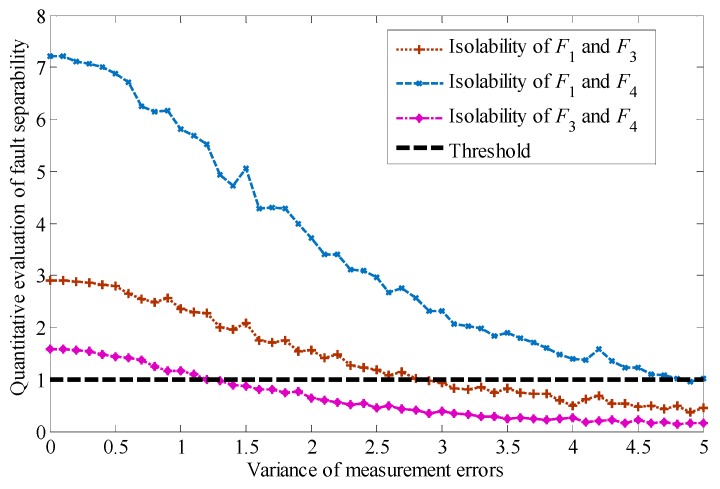
Quantitative evaluation of fault isolability under measurement errors.

Since the isolability index of faults $F_{1}$, $F_{3}$, and $F_{4}$ are the minimum value of fault diagnosability, we analyze the permissible area of measurement errors based on the isolability evaluation for these three kinds of faults. The results are shown in Figure 8. 

### 4.6. Permissible Area Analysis of Measurement Errors under Small Faults

As can be seen from Figure 7, an increase of measurement errors will cause the residual curves to coincide, which leads to a low fault diagnosability evaluation index of the system. However, more seriously, when the fault is a minor one, this trend will become more obvious, and the diagnosability of the fault will become more difficult. As shown in Table 5, minor faults occur in the system.

**Figure 9:**
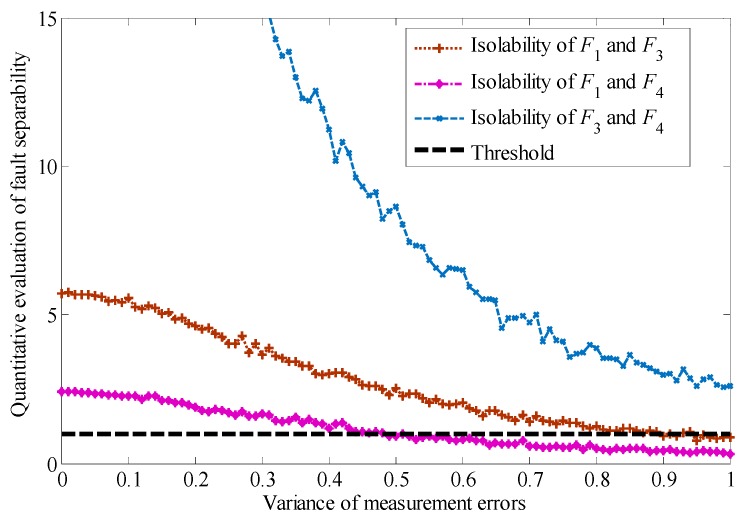
Quantitative evaluation of fault isolability under small faults.

In the presence of small faults, as shown in Table 5, the isolability evaluation index of faults is shown in Figure 9.

As can be seen from Figure 9, with a decrease of the fault, the residuals become smaller correspondingly, which leads to the further weakening of the isolability between faults $F_{1}$, $F_{3}$, and $F_{4}$. The isolability of faults can not be guaranteed when the variance is less than 0.5.

## 5. Conclusions

The detectability and isolability of faults are the preconditions for the fault diagnosis of control systems. However, the existence of measurement errors will make the quantitative evaluation index of fault diagnosability lower. In order to solve this problem, this paper proposes a design method for measuring the permissible area of the errors under the condition that the residuals obey the normal distribution. With the help of the KLD method, the permissible area of measurement errors is limited, and the accuracy of the quantitative evaluation of fault diagnosability is improved. The KLD method based on the similarity solution of the PDF can give accurate measurement errors within a feasible region even in the diagnosability evaluation of small faults because of its better sensitivity. It further provides a guarantee for improving the security and reliability of the system.

## Figures and Tables

**Table 1:** Process parameters and steady-state values of CSTRs.

Parameter	Value	Unit
*L*_0_, *L*_1_, *L*_3_	4.998, 4.998, 8	m^3^/h
*V*_1_, *V*_2_	1, 3	m^3^
*R*	8.314	kJ/kmol
*T*_0_, *T*_03_	280, 280	°C
*C*_A0s_, *C*_A03s_	2.4, 2.6	kmol/m^3^
*Q*_1s_, *Q*_2s_	0.7 × 10^6^, 0.3 × 10^6^	kJ/h
Δ*H*_1_	−1.00 × 10^5^	kJ/kmol
Δ*H*_2_	−1.04 × 10^5^	kJ/kmol
Δ*H*_3_	−1.08 × 10^5^	kJ/kmol
*k*10	3.0 × 10^6^	h^−1^
*k*20	3.0 × 10^5^	h^−1^
*k*30	3.0 × 10^5^	h^−1^
*E*1	5.0 × 10^4^	kJ/kmol
*E*2	7.53 × 10^4^	kJ/kmol
*E*3	7.53 × 10^4^	kJ/kmol
*ρ*	2000	Kg/m^3^
*c_p_*	0.731	kJ/kg
*T*_1s_, *T*_2s_	424.4, 444.5	°C
*C*_A1s_, *C*_A2s_	1.69, 0.89	kmol/m^3^

**Table 2:** Fault scenarios for reactors.

Fault	Steady-State	Faulty-State
*F*_1_: Faulty biased temperature sensor, *T*_0_	280	295–311 °C
*F*_2_: Faulty biased flow rate sensor, *L*_0_	4.998	5.25–5.5 m^3^/h
*F*_3_: Faulty biased temperature sensor, *T*_03_	280	295–311 °C
*F*_4_: Faulty biased flow rate sensor Fault, *L*_1_	4.998	5.25–5.5 m^3^/h

**Table 3:** Fault diagnosability evaluation results based on Kullback–Leibler divergence (KLD).

	*FD*	*F*_1_	*F*_2_	*F*_3_	*F*_4_
*F*_1_	611.12	0	244.90	2.7873	6.8912
*F*_2_	1787.2	140.57	0	113.00	99.419
*F*_3_	297.86	3.9086	290.31	0	1.4698
*F*_4_	171.06	19.898	520.73	2.5079	0

**Table 4:** Fault isolability evaluation results based on KLD.

		*σ*_0_ = 0.5	*σ*_0_ = 0.8	*σ*_0_ = 1.0	*σ*_0_ = 1.2	*σ*_0_ = 1.5	*σ*_0_ = 2.0	*σ*_0_ = 2.5	*σ*_0_ = 3.0
*μ*_0_ = 0	*F*_1_, *F*_2_	242.15	222.57	217.69	195.48	175.26	142.78	105.80	83.24
	*F*_1_, *F*_3_	2.7369	2.4951	2.4223	2.1212	1.9807	1.5732	1.2472	1.0294
	*F*_1_, *F*_4_	6.8078	6.1656	5.8939	5.3261	4.8636	3.8083	2.8577	2.3934
	*F*_2_, *F*_3_	115.58	106.82	105.35	97.181	89.689	75.715	64.867	58.227
	*F*_2_, *F*_4_	101.57	93.989	92.834	85.268	78.526	66.592	57.273	51.215
	*F*_3_, *F*_4_	1.4537	1.2418	1.0793	1.0213	0.8855	0.5855	0.4156	0.3502
*μ*_0_ = 0.5	*F*_1_, *F*_2_	247.39	230.02	210.99	197.73	175.50	140.79	107.76	90.059
	*F*_1_, *F*_3_	2.7955	2.6302	2.4247	2.2790	1.9507	1.6353	1.0568	1.0038
	*F*_1_, *F*_4_	6.9525	6.4617	5.8849	5.4837	4.7707	3.7963	2.7623	2.3265
	*F*_2_, *F*_3_	112.47	107.73	111.50	101.39	92.816	75.652	68.851	53.169
	*F*_2_, *F*_4_	98.809	94.574	97.920	89.249	80.772	66.774	60.050	46.841
	*F*_3_, *F*_4_	1.4492	1.3003	1.1656	0.9804	0.8439	0.5628	0.4744	0.3251

**Table 5:** Small fault scenarios for reactors.

Fault	Steady-State	Faulty-State
*F*_1_: Faulty biased temperature sensor, *T*_0_	280	280.5–281 °C
*F*_2_: Faulty biased flow rate sensor, *L*_0_	4.998	5.0–5.15 m^3^/h
*F*_3_: Faulty biased temperature sensor, *T*_03_	280	280.5–281 °C
*F*_4_: Faulty biased flow rate sensor Fault, *L*_1_	4.998	5.0–5.15 m^3^/h
